# ITK-targeted immune remodeling enhanced the efficacy of anti-CD19 CAR-T cell therapy

**DOI:** 10.1038/s41420-026-03004-2

**Published:** 2026-03-06

**Authors:** Zhenjun Li, Liangcheng Lv, Xiaoyu Yao, Zhiwei Feng, Yan Xie, Kecheng Li, Feifei Qi, Mei Yang, Jingwen Wang, Tao Pan, Xinghua Li, Haiyan Chen, Jing Wang, Yanping Ding, Jun Zhu, Yuqin Song, Xiaomin Wang, Ning Ding

**Affiliations:** 1 Key laboratory of Carcinogenesis and Translational Research (Ministry of Education), Laboratory of Lymphoma Translational Research, Peking University Cancer Hospital & Institute, Beijing, China; 2 Key laboratory of Carcinogenesis and Translational Research (Ministry of Education), Department of Lymphoma, Peking University Cancer Hospital & Institute, Beijing, China; 3 Angel Pharmaceuticals Co. Ltd, Jiaxing, Zhejiang Province China; 4 Beijing Imunopharm Technology Co. Ltd, Beijing, China

**Keywords:** Haematological cancer, Immunotherapy

## Abstract

Despite the promising efficacy of anti-CD19 CAR-T cells in treating B-cell lymphoma, T cell dysfunction and exhaustion remains a critical barrier to achieving durable responses. Based on the immunomodulatory effect in the clinical trial enrolled lymphoma patients, this study aims to explore the potential of the first in class highly selective ITK inhibitor soquelitinib in enhancing the persistence and antitumor functionality of CAR-T cells. We employed flow cytometric analysis to characterize T cell populations, RNA sequencing for gene expression profiling, and tumor bearing mice models to evaluate therapeutic efficacy. Our results demonstrated that the cytotoxic and anti-tumor activities of CAR-T cells were significantly increased post ITK inhibition treatment through elevation of cytotoxic and effector molecules, such as GZMB, TNF-α and IFN-γ. Meanwhile, soquelitinib promoted the expansion of CD8^+^ naïve and effector T cells while preventing exhaustion, as indicated by the downregulation of exhaustion markers such as TIM3, LAG3, and PD-1. Additionally, ITK inhibitor-treated CAR-T cells also exhibited increased cytotoxicity against malignant B cells and prolonged survival in tumor-bearing mice. Importantly, the modulation of transcription factors like TOX and TCF1 suggested a delay in T cell exhaustion and maintenance of effector functions. These findings provide a compelling rationale for the integration of clinical stage ITK inhibitor soquelitinib with CAR-T therapy, highlighting its potential to improve treatment outcomes in hematological malignancies and solid tumors.

## Introduction

Chimeric Antigen Receptor T-cell therapy (CAR-T therapy) was an innovative immunotherapy that modified patients’ T cells to eliminate tumor cells [1]. The anti-CD19 CAR-T therapy had been successfully used in treating blood cancers like B cell lymphoma and acute lymphoblastic leukemia (ALL) [2–4]. But it still comes with certain issues for a long-term clinical response [5, 6]. One of the issues was T cell exhaustion in the tumor microenvironment, as well as the limited capacity of CAR-T cells for long-term proliferation [7]. The effectiveness of CAR-T therapies was influenced by exhaustion, as exhausted T cells exhibited limited proliferation activity, lower cytokine production, and impaired cytotoxicity, potentially leading to treatment failure [8].

T cell exhaustion was the dysfunction status that developed during chronic infections and cancer, characterized by a progressive loss of T cell effector functions – including the production of cytokines and cytotoxic activity [9]. This condition occurred mainly due to the exposure to antigens for extended periods, which induced the expression of inhibitory receptors, such as PD-1, TIM3, and LAG3 [10, 11]. During T cell exhaustion process, the related signaling pathways were altered and transcription factors, such as EOMES, TOX and T-bet became activated [12–14]. T cell exhaustion hindered the clinical efficacy of CAR-T therapy in cancer, especially B-cell malignancies. Even though CAR-T therapies were initially successful, patients often relapsed because the CAR-T cells were unable to persist and/or function effectively. The current strategies for T cell exhaustion were limited and mostly involved immune checkpoint blockade or combination therapies. Nevertheless, these strategies did not fully address the fundamental mechanisms of exhaustion. As a result, there is considerable gap of research that might help enhance long-term persistence and functionality of CAR-T cells within tumor microenvironment [15].

Many studies had highlighted the importance of different molecular targets that affected T cell exhaustion, especially epigenetic modification and intracellular signal regulators within T cells [16, 17]. Interleukin-2-inducible T-cell kinase (ITK) was essential for T cell receptor signaling and regulated T cell activation, differentiation, and survival [18, 19]. It was reported that chronic activation of the TCR signaling pathway contributed to T cell exhaustion [20]. Evidence from mice models showed that blocking ITK activity prevented T cell exhaustion [21]. Blocking ITK activity had shown the potential in boosting T cell responses and preventing exhaustion, making it a useful target for improving CAR-T therapy efficiency. Nevertheless, the molecular mechanisms by which ITK inhibition affected T cell activity and improved CAR-T cell function are still poorly understood. More studies are still needed to clarify how ITK inhibition enhances T cell functionality and further explore potential combination treatment strategies.

Soquelitinib, the first in class highly selective ITK inhibitor, characterized the dissociation constant (Kd) of 6.5 nM and demonstrated over 80-fold selectivity relative to other cysteine-containing kinases, which had undergone global phase I/Ib clinical trials [22]. In this study, we investigated the immunomodulatory effect of soquelitinib on T cell populations, which enhanced anti-tumor activity of CAR-T cells against B-cell malignancies. Through the application of flow cytometric analysis, RNA sequencing, and tumor-bearing mice models, our study clarified the impact of ITK inhibition in CAR-T cell proliferation, functionality and exhaustion. Our results revealed the valuable insights into new therapeutic approaches to address T cell exhaustion, which may improve the treatment outcomes of hematological malignancies and solid tumors patients.

## Results

### Blocking ITK potentiates CAR-T cytotoxicity

**Fig. 1:**
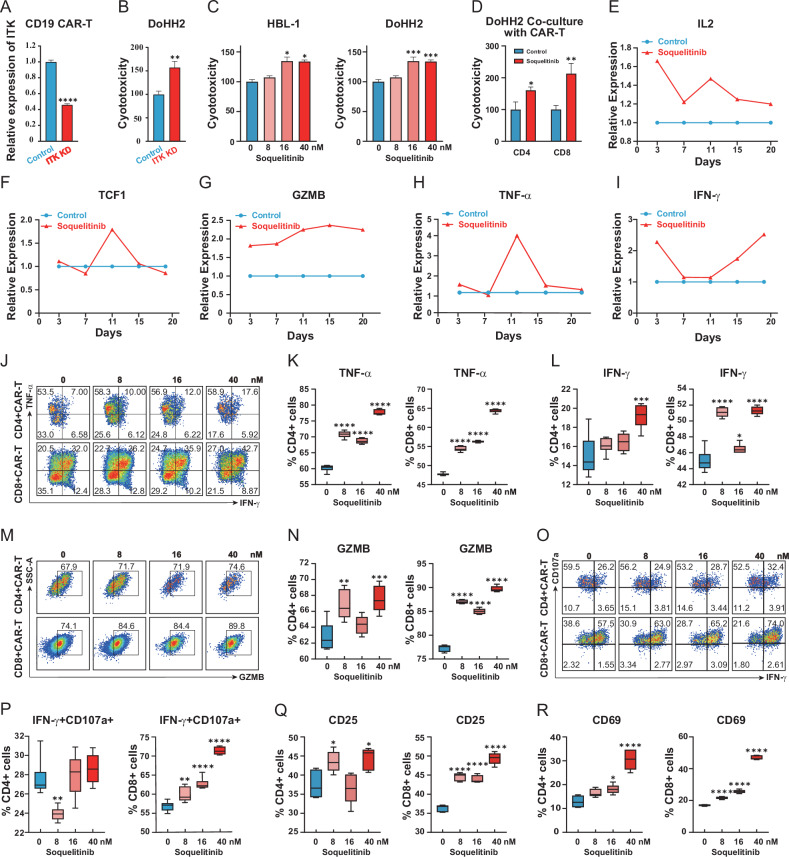
ITK Inhibition enhance CD19 CAR-T cell activation and cytotoxic function. **A** ITK mRNA expression in CD19 CAR-T cells with or without ITK shRNA knockdown (RT-qPCR; normalized to GAPDH); *n* = 4. **B** Enhanced cytotoxicity of ITK-knockdown (KD) CD19 CAR-T cells against DoHH2 lymphoma cells. Coculture duration: 6 h; E:T ratio=1:1; *n* = 5. **C** Dose-dependent potentiation of cytotoxicity by soquelitinib (0–40 nM) in CD19 CAR-T cells against B-cell lymphomas (HBL-1, DoHH2). Coculture duration: 6 h; E:T ratio=1:1; *n* = 4–5. **D** CAR-T cells were treated with soquelitinib or vehicle control, after which CD4^+^ and CD8^+^ subsets were isolated using fluorescence-activated cell sorting (FACS). The CD4^+^ and CD8^+^ subsets were then individually assessed for their cytotoxic activity against DoHH2 target cells. Coculture duration: 6 h; E:T ratio=4:1; *n* = 3-4. **E**–**I** Soquelitinib (40 nM)-mediated upregulation of effector genes: IL-2, TCF1, GZMB, TNF-α, and IFN-γ (RT-qPCR; normalized to GAPDH); *n* = 5. **J** Representative flow cytometry contour plots of TNF-α^+^ and IFN-γ^+^ cells in CD4⁺ (upper) and CD8⁺ (lower) CAR-T subpopulations. **K** Quantification of TNF-α^+^ cells in CD4^+^ and CD8^+^ subsets; *n* = 6. **L** Quantification of IFN-γ^+^ cells in CD4^+^ and CD8^+^ subsets; *n* = 6. **M** Representative flow cytometry contour plots of GZMA^+^ cells in CD4⁺ (upper) and CD8⁺ (lower) CAR-T subpopulations. **N** Quantification of GZMA^+^ cells in CD4^+^ and CD8+ subsets; *n* = 5. **O** Representative flow cytometry contour plots of IFN-γ^+^CD107a^+^ cells in CD4⁺ (upper) and CD8⁺ (lower) CAR-T subpopulations. **P** Quantification of IFN-γ^+^CD107a^+^ cells in CD4^+^ and CD8^+^ subsets; *n* = 6. **Q** Quantification of CD25^+^ cells in CD4^+^ and CD8^+^ subsets; *n* = 5. **R** Quantification of CD69^+^ cells in CD4^+^ and CD8^+^ subsets; *n* = 5.

To delineate the mechanistic role of ITK signaling in constraining CAR-T cell effector function, we integrated genetic ablation (shRNA) and pharmacologic inhibition (soquelitinib), coupled with multi-parametric functional characterization spanning cytotoxicity, transcriptional regulation of select effectors, and targeted protein analysis. ITK knockdown potentiated CD19 CAR-T cell-mediated cytotoxicity against B lymphoma cells (DoHH2), as evidenced by enhanced target cell lysis in LDH release assays (Fig. 1A, B). Pharmacologic ITK inhibition using the clinical-stage inhibitor soquelitinib ( > 80-fold cysteine kinase selectivity) recapitulated this potentiation, achieving dose-dependent augmentation of CAR-T cell cytotoxicity against B-cell lymphomas (HBL-1 and DoHH2, Fig. 1C). To pinpoint the cellular subset mediating the enhanced cytotoxicity, we sorted CD4^+^ and CD8^+^ CAR-T cells after soquelitinib treatment. Soquelitinib significantly boosted the killing capacity of CD8^+^ CAR-T cells and CD4^+^ CAR-T cells against DoHH2 and HBL-1 cells (Fig. 1D and Supplementary Fig. 1A). Mechanistically, soquelitinib treatment induced coordinated changes in CAR-T cell function, concomitant with upregulation of effector cytokines (TNF-α, IFN-γ and IL-2), cytolytic factor GZMB, and memory-associated transcription factor TCF1, evidenced by increased transcript (Fig. 1E–I) and protein expression levels (Fig. 1J–N). Furthermore, we found that soquelitinib treatment enhanced the degranulation capacity of CAR-T cells, thereby enhancing CD107a expression (Fig. 1O, P). This functional potentiation was further validated by elevated expression of early activation markers CD25 and CD69 across CD4^+^ and CD8^+^ CAR-T subsets (Fig. 1Q, R and Supplementary Fig. 1B, C), suggesting ITK blockade enhanced the cytotoxic potential of CAR-T cells.

### Soquelitinib enhances CAR-T fitness through enhanced expansion, survival and cytokine output

**Fig. 2:**
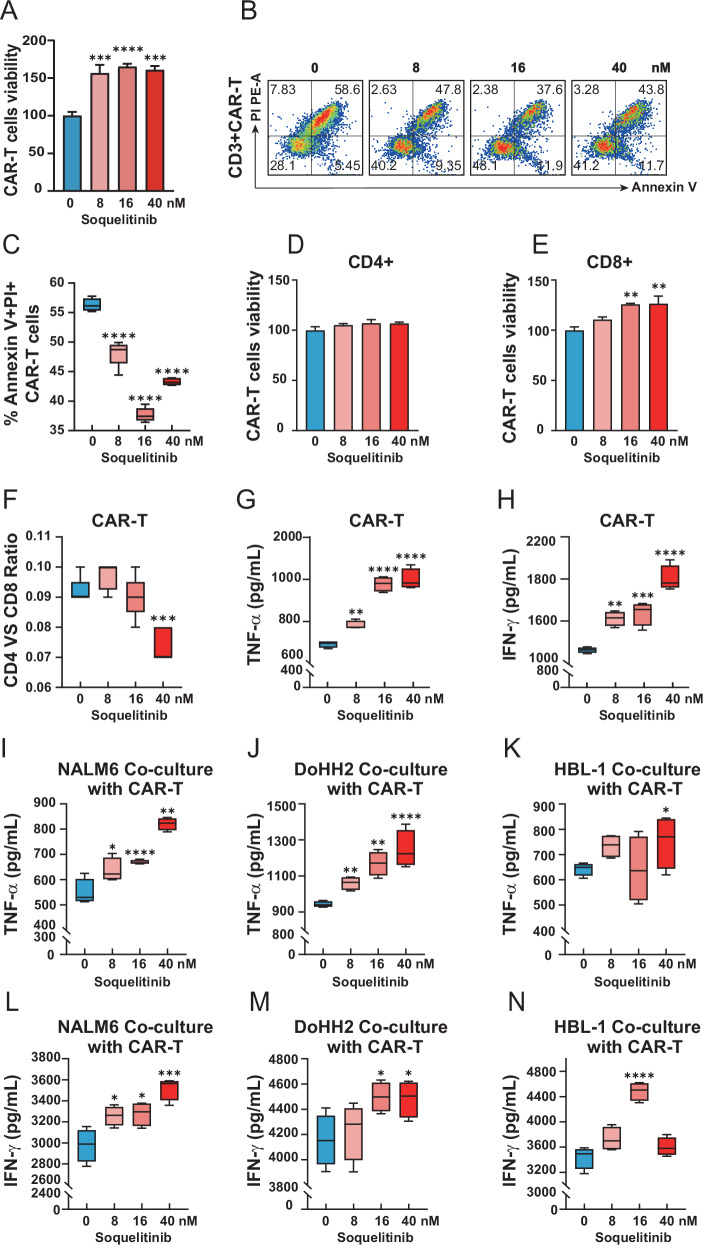
Soquelitinib enhances the expansion, long-term persistence, and antitumor cytokine secretion capacity of CD19-CAR-T cells in vitro. **A** Dose-dependent potentiation of total CD19 CAR-T cell expansion by soquelitinib (0–40 nM) during CD3/CD28 activation (7-day culture); *n* = 4. **B** Annexin V/PI flow cytometry contour plots demonstrating reduced apoptosis with ITK inhibition (20-day culture). **C** Quantification of late apoptotic cells (Annexin V^+^ PI^+^); *n* = 6. **D**, **E** The viability of CD8^+^ CAR-T cells and CD4^+^ CAR-T cells; *n* = 3. **F** The ratio of CD4^+^CAR-T/CD8^+^CAR-T was analyzed before and after soquelitinib treatment; *n* = 5. **G**, **H** Soquelitinib elevated TNF-α and IFN-γ production in PMA/ionomycin-stimulated CAR-T cells; *n* = 4. **I**–**N** Soquelitinib elevated TNF-α and IFN-γ production in tumor antigen-specific stimulated CAR-T cells; *n* = 4.

To investigate the functional impact of ITK inhibition on CAR-T cell fitness, we administered soquelitinib treatment during CAR-T cell activation and assessed proliferation, viability and cytokine production. The ex vivo expansion of CD19 CAR-T cells was significantly enhanced by soquelitinib, as evidenced by increased viability and reduced apoptosis over a 20-day culture period (Fig. 2A–C). CD8^+^ CAR-T subsets had a more robust proliferation potentiation on ITK inhibition compared to CD4^+^ cells (Fig. 2D, E). Meanwhile, soquelitinib treatment dramatically decreased the CD4/CD8 ratio by flow cytometry (Fig. 2F). Soquelitinib increased production of TNF-α and IFN-γ in CAR-T cells following PMA and ionomycin stimulation (Fig. 2G, H). Significantly, we also found that this enhancement also applied to tumor antigen-specific stimulation which was seen in the significantly elevated production of TNF-α and IFN-γ production upon coculture with NALM6, DoHH2, and HBL-1 lymphoma cells (Fig. 2I–N). Overall, ITK blockade with soquelitinib enhanced CAR-T cell function through increased proliferation capacity, decreased apoptosis and enhanced cytokine production observed in both pharmacologic (PMA/ionomycin) or tumor antigen-specific stimulation context.

### Soquelitinib polarizes CAR-T cell differentiation to boost CD8^+^ cytotoxicity

**Fig. 3:**
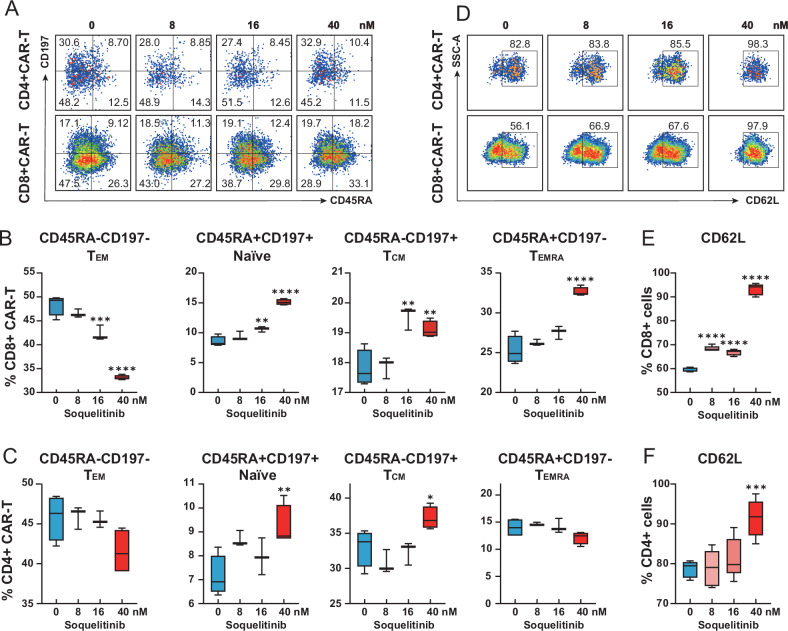
Soquelitinib reprograms CAR-T cell differentiation. **A** Flow cytometric gating strategy for CD19 CAR-T cell subsets after 7-day exposure to soquelitinib (0-40 nM) with anti-CD3/CD28 bead activation and IL-2 (500 IU/mL). Subsets defined by: Naïve, CD45RA^+^CD197^+^; TCM, central memory, CD45RA^−^CD197^+^; TEM, effector memory, CD45RA^-^CD197^-^; TEFF, CD45RA^+^CD197^−^. **B**, **C** Soquelitinib dose-dependent modulation of CD8^+^ CAR-T cell subsets and CD4^+^ CAR-T cell subsets; *n* = 3, 4. **D** Representative histograms of CD62L expression (L-selectin) in CD4^+^ and CD8^+^ CAR-T cells. **E**, **F** Soquelitinib dose-dependent modulation of CD62L^+^ CD8^+^ and CD62L^+^ CD4^+^ CAR-T cells; *n* = 5.

To further evaluate the modulatory effects of ITK inhibition on CAR-T cell differentiation we profiled T-cell functional immunophenotypes via flow cytometry after exposure to soquelitinib. In vitro activation of CD19 CAR-T cells was performed with anti-CD3/CD28 beads and IL-2 stimulation (100 IU/mL). Soquelitinib treatment changed the landscape of T cell differentiation in a dose-dependent manner, according to the surface marker analysis. In CD8^+^ CAR-T cells, (1) Naïve T cells (CD45RA^+^ CD197^+^) increased from 8.53% to 15.14% (*P* < *0.0001*); (2) Central memory T cells (CD45RA^-^ CD197^+^) rose from 17.80% to 19.10% (*P* < *0.01*); and (3) Terminal effector memory T cells (TEMRA cells, CD45RA^+^ CD197^-^) expanded from 25.29% to 32.63% (*P* < *0.0001*) (Fig. 3A, B). In CD4^+^ CAR-T cells, (1) Naïve T cells increased from 7.14% to 9.22% (*P* < *0.01*); (2) Central memory T cells elevated from 33.04% to 37.14% (*P* < *0.05*) (Fig. 3A, C). As CD62L (L-selectin) served as a canonical regulator of lymphocyte homing and memory differentiation, we quantified its expression on CAR-T cells by flow cytometry post-soquelitinib exposure. Notably, soquelitinib significantly increased the frequency of CD62L^+^ cells within CD8^+^ CAR-T cells from 59.47% to 93.18% (*P* < *0.0001*; 1.57-fold) and within CD4^+^ CAR-T cells from 78.66% to 91.43% (*P* < *0.001*; 1.16-fold) at 40 nM relative to untreated controls (Fig. 3D–F). Taken together, ITK inhibition by soquelitinib reprogramed CAR-T cell differentiation, manifesting as a selective enhancement of CD8^+^ cytotoxic efficacy.

### ITK inhibition reprograms transcriptional networks to rescue CAR-T cells from exhaustion

**Fig. 4:**
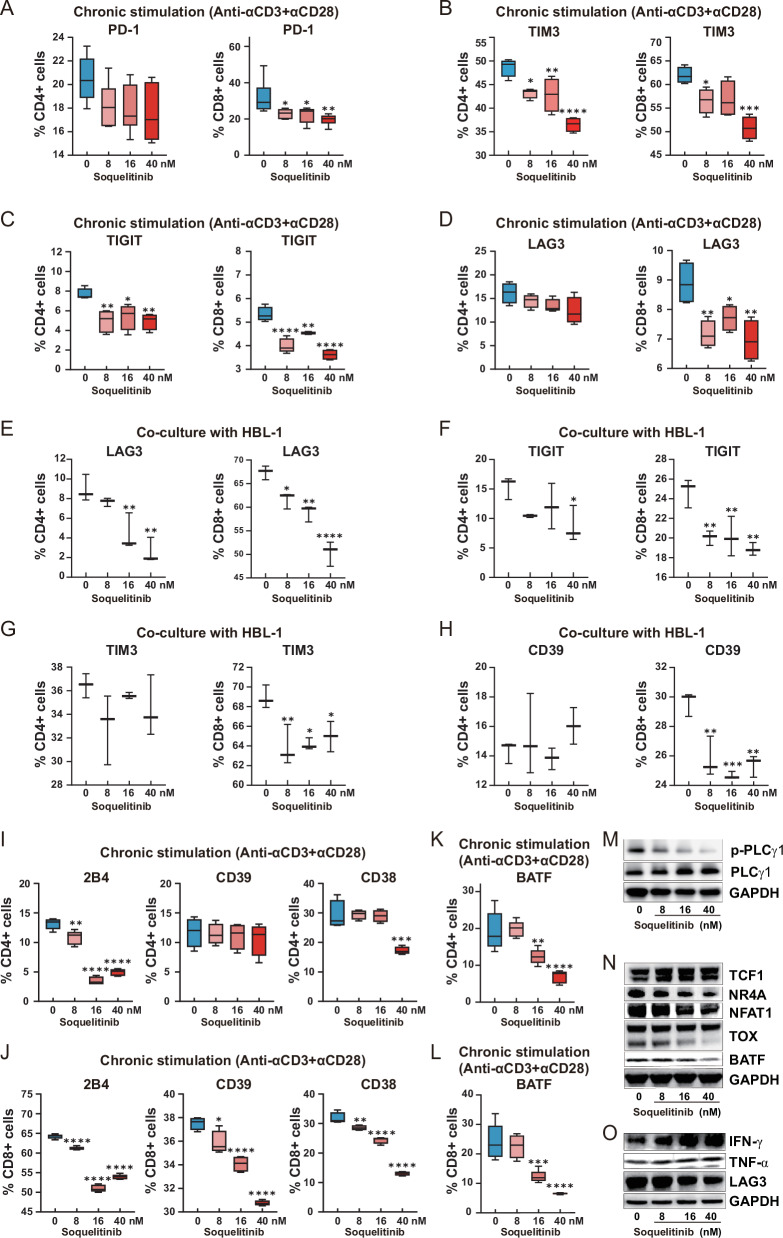
ITK inhibitor soquelitinib mitigates exhaustion in CD19 CAR-T cells in vitro. **A**–**D** Summary data showing the percentages of PD-1^+^, TIM3^+^, TIGIT^+^, and LAG3^+^ cells within CD4^+^ and CD8^+^ CD19 CAR-T cell populations after 7-day exposure to soquelitinib (0-40 nM) with anti-CD3/CD28 bead activation and IL-2 (500 IU/mL); *n* = 4–6. **E**–**H** Summary data showing the percentages of LAG3^+^, TIGIT^+^, TIM3^+^, and CD39^+^ cells within CD4^+^ and CD8^+^ CD19 CAR-T cell populations after 7-day exposure to soquelitinib (0–40 nM). CD19 CAR-T cells were co-cultured with HBL-1 tumor cells and subjected to repetitive antigen stimulation over a 7-day period; *n* = 3. **I**, **J** Summary of percentages of 2B4^+^, CD39^+^ and CD38^+^ cells within CD4^+^ and CD8^+^ CD19 CAR-T cell populations; *n* = 4–5. **K**, **L** The expression of nuclear transcription factors BATF was analyzed within CD4^+^ and CD8^+^ CAR-T cells by flow cytometry; *n* = 5. **M** The expression of p-PLCγ-1and PLCγ-1 were analyzed by western blotting (GAPDH used as loading control). **N** The expression of transcription factors TCF1, NR4A, NFAT1, TOX and BATF were analyzed by western blotting (GAPDH used as loading control). **O** The expression of IFN-γ, TNF-α and LAG3 were analyzed by western blotting (GAPDH used as loading control).

T cell exhaustion critically compromised CAR-T cell efficacy by enforcing dysfunctional transcriptional circuits. To investigate whether ITK blockade via soquelitinib intercepted this process, we dissected its impact on exhaustion circuitry through quantification of key regulators mechanistically linked to CAR-T cell dysfunction. CD19 CAR-T cells were activated with anti-CD3/CD28 beads + IL-2 (500 IU/mL) with or without soquelitinib (0–40 nM) for 7 days. Flow cytometry quantification revealed potent suppression of exhaustion markers by soquelitinib (40 nM) (Fig. 4A–D and Supplementary Fig. 2A–D): (1) in CD8^+^ CAR-T cells, PD-1 decreased from 31.86% to 19.59% (*P* < *0.01*), TIGIT decreased from 5.33% to 3.62% (*P* < *0.0001*), TIM3 decreased from 61.95% to 50.78% (*P* < *0.001*), and LAG3 decreased from 8.90% to 6.95% (*P* < *0.01*); (2) in CD4^+^ CAR-T cells, TIGIT decreased from 7.67% to 4.93% (*P* < *0.01*) and TIM3 decreased from 47.93% to 37.98% (*P* < *0.0001*). Additionally, we have performed an experiment using an antigen-specific cells repeated stimulation model to assess the effect of soquelitinib on CAR-T cell exhaustion. Specifically, we co-cultured CD19-targeting CAR-T cells (with or without soquelitinib treatment) with HBL-1 tumor cells and performed repeated antigen challenges over an extended period to establish a long-term exhaustion model. The results showed that soquelitinib treatment significantly decreased the expression of key exhaustion markers (TIM3, TIGIT, LAG3, and CD39) on CD8^+^ and CD4^+^ CAR-T cells even after multiple rounds of antigen-specific stimulation with HBL-1 cells (Figure 4E–H and Supplementary Fig. 2E–H). Concurrently, soquelitinib (40 nM) significantly downregulated multifactorial drivers of exhaustion, including the coinhibitory receptor 2B4, metabolic regulators CD38, and transcription factor BATF in CD8^+^ and CD4^+^ CAR-T cells, metabolic regulators CD39 in CD8^+^ CAR-T cells, compared to untreated controls (*P* < *0.001* for all; Fig. 4I–L and Supplementary Fig. 2I–L). To confirm the on-target inhibition of ITK, we assessed the phosphorylation of its key downstream effector, PLCγ-1. Western blot analysis revealed that soquelitinib treatment significantly reduced PLCγ-1 phosphorylation in activated CAR-T cells (Fig. 4M), confirming the effective blockade of the ITK signaling pathway. Western blot analyses demonstrated that soquelitinib (40 nM) profoundly reprogramed the transcriptional landscape of CAR-T cells, suppressing exhaustion-associated transcription factors NR4A, TOX, NFAT1, and BATF, while elevating the memory regulator TCF1 (Fig. 4N). Consistent with enhanced CAR-T cell activation via ITK inhibition, it significantly enhanced TNF-α and IFN-γ protein expression (Fig. 4O). In summary, soquelitinib-mediated ITK blockade rescued CAR-T cells from exhaustion by dismantling the NFAT-driven transcriptional circuitry, simultaneously reversing inhibitory receptor expression in CD8^+^ CAR-T cells.

### Soquelitinib augments CD19 CAR-T cell-mediated eradication of B cell malignancy via ITK inhibition

**Fig. 5:**
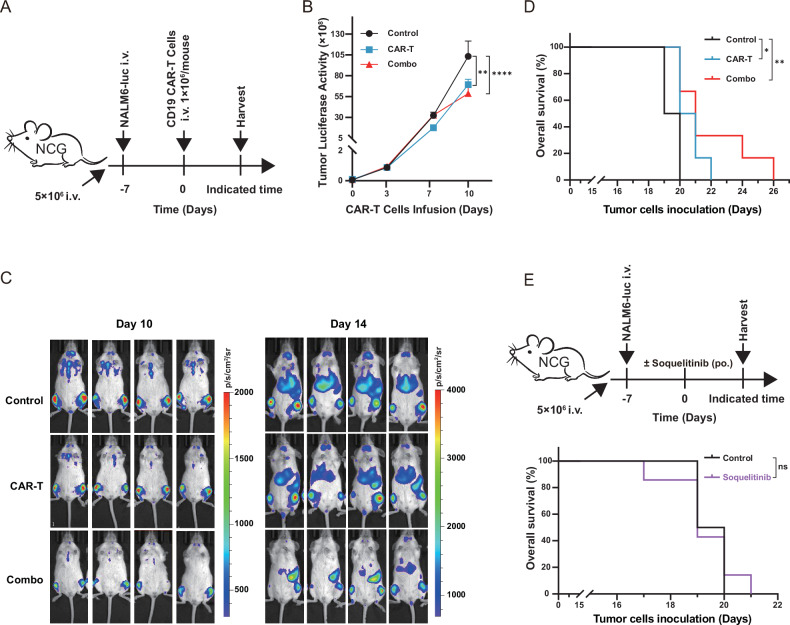
Soquelitinib enhances CD19 CAR-T cell efficacy in ALL mouse models. **A** NCG mice received intravenous injection of luciferase-expressing NALM6 cells (5 × 10⁶ cells/mouse) on Day (−7) to establish ALL mouse models. Anti-CD19 CAR-T cells (2 × 10⁶ cells/mouse) were infused ± soquelitinib (30 mg/kg, oral gavage daily) on Day 0. **B** Tumor burden quantification: Bioluminescent flux (scale: 10⁸ photons/sec/cm²/sr) measured in regions of interest (ROI) following IVIS imaging at indicated timepoints; *n* = 6. **C** Representative in vivo bioluminescence: Images visualize the marked attenuation of tumor signal in the combination group on Day 10 (scale: photons/sec/cm²/sr) and Day 14 (scale: photons/sec/cm²/sr). **D** Survival analysis: Kaplan-Meier curves demonstrate prolonged survival in the combination group; *n* = 6. **E** NCG mice received intravenous injection of luciferase-expressing NALM6 cells (5 × 10⁶ cells/mouse) on Day (−7) to establish ALL mouse models. These mice were treated with either soquelitinib (30 mg/kg, oral gavage daily) or vehicle control, starting on Day 0, without any CAR-T cell infusion. Kaplan-Meier analysis revealed no significant survival benefit with soquelitinib treatment compared to the control group; *n* = 6–7.

To delineate the in vivo efficacy of ITK inhibitor soquelitinib in enhancing CAR-T cell function, we engrafted luciferase-expressing NALM6 cells into immunodeficient mice to establish disseminated B cell tumor-bearing mice models. Anti-CD19 CAR-T cells were infused intravenously 7 days post tumor cells engraftment (Fig. 5A). Strikingly, the soquelitinib co-administration synergized with CAR-T therapy to more effectively suppress tumor progression, manifesting as a 2.10 × 10^9^ and 2.32 × 10^9^ reduction in bioluminescent flux at 10 and 14 days post CAR-T infusion (Fig. 5B, C), while extending survival range from 22 to 26 days (Fig. 5D). Soquelitinib exhibited no significant anti-tumor activity compared to the vehicle control group, as assessed by both bioluminescent imaging of tumor burden and overall survival analysis (Fig. 5E and Supplemenry Fig. 3A). These data establish ITK blockade as a clinically actionable strategy to optimize adoptive T-cell therapies.

### ITK Inhibition Reprograms Transcriptional Circuitry to Potentiate Effector Function and Counteract Exhaustion in CAR-T Cells

**Fig. 6:**
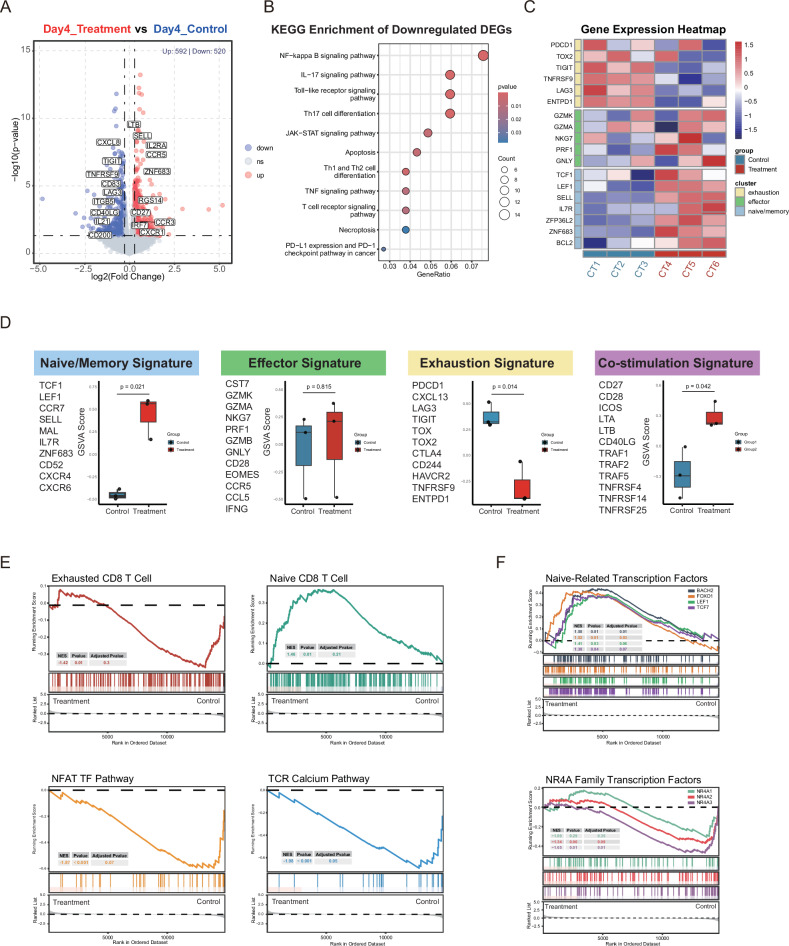
The Transcriptional Impact of ITK Inhibitor on CD19 CAR-T Cells. **A** Volcano plots of genes differentially expressed in CD19 CAR-T cells compared with ITK inhibitor treated CD19 CAR-T cells. Selected differentially expressed genes with an adjusted *P*-value ≤ 0.05 and log2 fold change > 0.28 or −0.28 are indicated. **B** KEGG analysis of the downregulated differentially expressed genes. **C** Heat maps showing expression of representative genes in CD19 CAR-T cells vs. ITK inhibitor treated CD19 CAR-T cells in individual replicates. **D** The box plots demonstrating GSVA score differences between CD19 CAR-T cells and ITK inhibitor treated CD19 CAR-T cells across selected gene sets, with each gene set labeled on the left side of the corresponding plot. **E** GSEA of RNA-seq data from CD19 CAR-T cells and ITK inhibitor treated CD19 CAR-T cells displayed as enrichment plots, ranking genes by fold change in expression between those conditions. ( | NES | > 1, *P* value < 0.05, FDR < 0.25). **F** GSEA analysis of specific transcription factor activities in CAR-T cells from the ITK inhibitor treatment and control groups based on the CHEA database dataset. (*n* = 3/group).

To characterize ITK inhibition-mediated transcriptional reprogramming in CD19 CAR-T cells, soquelitinib (40 nM) or vehicle control was administered during T cell activation (CD3/CD28 antibodies + IL-2 [500 IU/mL]) for 4 days prior to bulk RNA-seq analysis. We identified 1,112 differentially expressed genes (DEGs) ( | FC | ≥ 1.2, *P* < 0.05), comprising 592 upregulated and 520 downregulated transcripts (Fig. 6A). KEGG enrichment analysis demonstrated that soquelitinib significantly reduced TCR, NF-kB, and PD-L1/PD-1 signal pathway in CD19 CAR-T cells (Fig. 6B). Furthermore, transcriptional profiling further showed that the genes associated with T cell effector functions—including GZMK, GZMA, NKG7, and PRF1—were upregulated in ITK inhibitor-treated CD19 CAR-T cells. Additionally, we observed upregulation of genes related to naïve or progenitor-like memory T cells, including TCF1, IL7R, SELL, LEF1, and ZNF683. Conversely, genes involved in T cell exhaustion, such as PDCD1 (encoding PD-1), TIGIT, TOX, and LAG3, were downregulated (Fig. 6C). Gene Set Variation Analysis (GSVA) scoring results were consistent with the above findings, showing upregulated naïve /memory signature (*P* = 0.021), co-stimulation signature (*P* = 0.042) effector signature (*P* = 0.815) and downregulated exhaustion signature (*P* = 0.014) in soquelitinib treated CD19 CAR-T cells (Fig. 6D). Gene Set Enrichment Analysis (GSEA) revealed a negative enrichment in exhausted CD8^+^ T cells related genes, (NES = -1.43, *P* = 0.012), NFAT transcription factor pathway (NES = −1.88, *P* = 0.00044), and the TCR calcium pathway (NES = −1.98, *P* = 0.00015). Conversely, a positive enrichment was observed for naïve CD8^+^ T cells related genes (NES = 1.46, *P* = 0.0063) in soquelitinib-treated CD19 CAR-T cells (Fig. 6E). Using ChEA Transcription Factor Targets Dataset, we analyzed transcription factor activity of CAR-T cells post ITK inhibitor treatment. The analysis revealed a significant decrease in NR4A family activity, while transcription factors linked to T cell stemness, such as FOXO1, TCF1, BACH2, and LEF1, were significantly increased (Fig. 6F). Taken together, inhibiting ITK could reduce CD19 CAR-T cell exhaustion, enhance effector function, and promote memory formation.

## Discussion

**Fig. 7:**
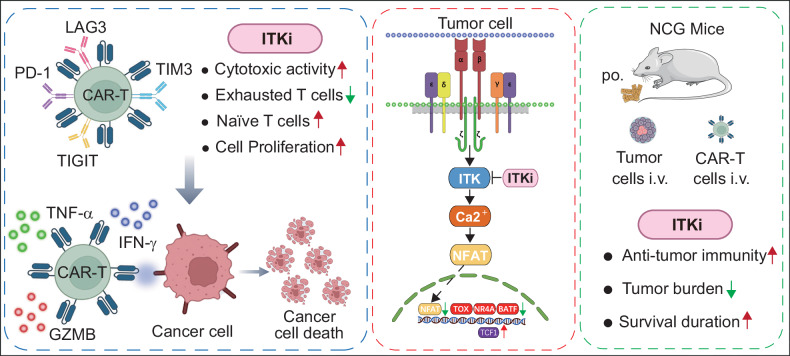
Proposed model on the role of ITK inhibition in CAR-T cell exhaustion and function. Left: Exhausted CAR-T cells exhibit high expression of exhaustion markers such as PD-1, LAG3, TIM3, and TIGIT. Treatment with an ITK inhibitor reduces the expression of these exhaustion markers and increases the production of effector molecules, including TNF-α, IFN-γ, and granzyme B (GZMB. ITK inhibitor-treated CAR-T cells show an increased proportion of Naïve T cells, a decreased proportion of exhausted T cells, enhanced proliferation, and improved cytotoxic activity. Central: The ITK inhibitor attenuates nuclear translocation of NFAT, leading to downregulation of exhaustion-associated transcription factors (e.g., TOX, NR4A, BATF) and upregulation of the memory-associated transcription factor TCF1. Right: NCG mice were inoculated with NALM6-luc cells via tail vein injection and treated with CD19 CAR-T cells. Compared to CD19 CAR-T cell monotherapy, the combination of an ITK inhibitor and CD19 CAR-T cells enhanced antitumor efficacy, as evidenced by reduced tumor burden and prolonged survival.

Understanding T cell exhaustion was essential for improving the outcome of cancer treatment, especially in CAR-T therapy for B-cell malignancies [23]. The currently approaches for the treatment of T cell exhaustion had limited success, revealing a significant gap in research focused on enhancing the functionality of CAR-T cells. In the present work, we had efficiently prioritized and delineated a consistent mechanistic pathway by which ITK inhibition provided beneficial effects. Our results provided strong evidence for the model that soquelitinib-mediated ITK inhibition reduced expression of key transcription factors that drive exhaustion (including NFAT1, TOX, and BATF). ITK inhibition led to a reduction in the surface levels of PD-1, TIM3, LAG3, and TIGIT, which boosted the efficacy of CAR-T cells against tumor cells in vitro and in vivo. Importantly, the RNA-seq results also indicated the suppression of genetic programs and signaling pathways associated with exhaustion, such as NFAT and TCR signaling pathway, during ITK inhibitor treatment process (Fig. 7).

Continuous and repetitive exposure to antigens and constant expression of inhibitory receptors, in addition to an immunosuppressive tumor microenvironment, were related to the dysfunction and exhaustion of tumor-infiltrating T cells. T cell exhaustion was characterized by over-expression of inhibitory receptors and reduced cytotoxicity activity. The use of immune checkpoint blockade had been effective to prevent T cell exhaustion in cancer immunotherapy [24]. Applying CAR-T cells to produce the PD-1/CTLA-4 antibodies or using CRISPR/Cas9 to remove the inhibitory receptors could enhance anti-tumor activity of CAR-T cells [25, 26]. Moreover, the immunosuppressive tumor microenvironment where DCs, monocytes, and CD4^+^ T-cells secreted the inhibitory soluble cytokines IL-10 and TGF-β also played a significant role in suppressing T-cell activity [27]. Additionally, immunoregulatory cells, such as myeloid-derived suppressor cells (MDSCs), produced soluble factors that included reactive oxygen species (ROS), inducible nitric oxide synthase (ion’s), and arginase-1 that hindered T-cell activation and accelerate T cell exhaustion [28]. The function of CAR-T therapy could also be inhibited by CD4^+^ regulatory T cells (Tregs), resulting in tumor recurrence and exhaustion of CD8^+^ T cells [29]. Based on these evidences, the blockade of IDO1 and VEGF as well as the usage of TGF-β-resistant CAR-T cells had exhibited enhanced anti-tumor efficacy in several preclinical and clinical models, emphasizing the need to target immunosuppression for improving CAR-T therapy [30, 31]. In our study, we demonstrated that prolonged exposure to tumor antigens led to chronic activation of CAR and TCR signaling pathways that resulted in T cell exhaustion. Nonetheless, this exhaustion was reduced upon the use of an ITK inhibitor, which correlated with enhanced anti-tumor effects in CD19 CAR-T therapy. The immunomodulatory effects of ITK inhibition on CAR-T cells also requires further validation in infused CAR-T cells from patients with B-cell lymphoma.

In this study, we investigate the potential of a novel ITK inhibitor to enhance the function of CAR-T therapy. Soquelitinib treatment inhibited the CD4/CD8 ratio, indicating a marked bias in favor of CD8^+^ T cells expansion and/or differentiation. Moreover, the flow cytometric results consistently showed that following the treatment of soquelitinib, the levels of cytotoxic and effector molecules (TNF-α, IFN-γ, and GZMB) in CD4^+^ and CD8^+^ CAR-T cell subsets were upregulated. TCF1, or T cell factor 1, was an important transcription factor in WNT signaling pathway, which played an important role in T cell development and immunity [32]. It was reported that TCF1 could help maintain stem-like characteristics in CD8^+^ precursor exhausted T cells, which were capable of self-renewal and differentiation [33]. Furthermore, previous studies had indicated that TCF1 may also repress TOX, a factor associated with T cell exhaustion, thereby maintaining T cell stem-like states [34]. This balance between T cell stemness and exhaustion was critical for the efficacy of immunotherapy. TCF1-activated CD8^+^ T cells increased the expression of effector molecules such as granzyme B, TNF, and IFN-γ through EOMES upregulation, thereby enhancing T cell anti-tumor activity [35]. The significance of these findings suggested that combining CAR-T cells with these immune-modulating targets could synergistically enhance anti-tumor immunity and extend the persistence of immunotherapy.

The modulation of key regulators such as TOX and NR4A by ITK inhibitor presented a promising therapeutic strategy for enhancing CAR-T cells efficacy. TOX was a transcription factor that belonged to the HMG-box superfamily [36]. The NR4A family consisted of the orphan nuclear receptors NR4A1, NR4A2, and NR4A3 [37]. The expression of these transcription factors regulated T cell tolerance, exhaustion, metabolism and inflammation process. They created a positive feedback loop where TOX and TOX2 increased NR4A expression, which in turn enhanced TOX expression, thus sustaining the exhausted status of T cells [38]. This network upregulated PD-1 (PDCD1), TIM3 (HAVCR2), LAG3, TIGIT, and CD244 while downregulating effector cytokines such as IFN-γ and TNF-α. Importantly, previous studies had shown that CAR-T cells deficient for NR4A expressed lower levels of these inhibitory receptors and produced more cytokines, which led to improved tumor regression in mouse models of solid tumors [39]. Moreover, we demonstrated that targeting of ITK could inhibit T cell exhaustion through down-regulation of the transcription factors TOX and NR4A. This study highlighted the significance of understanding these signaling dynamics and molecular mechanisms, as they could guide the development of combination therapies that target specific components of the immune response, ultimately enhancing patient outcomes in CAR-T therapy. Additionally, these findings also highlighted the importance of studying the pharmacokinetics and pharmacodynamics of ITK inhibitor in clinical settings to optimize therapeutic use.

In conclusion, this study highlighted the important role of ITK inhibition in enhancing the effectiveness of CAR-T cells against tumors by facilitating T cell growth and reducing exhaustion. The strong evidences provided supported the idea that incorporating ITK inhibitor could be a valuable approach to improve treatment results in both blood cancers and solid tumors. By tackling the significant issues related to T cell exhaustion, these findings opened up new possibilities for creating innovative combination therapies that could ultimately lead to more lasting and effective CAR-T cell treatments in clinical practice.

## Materials and methods

### Cell lines

The lymphoma cell lines HBL-1 and DoHH2 were kindly provided by Dr. Kai Fu, University of Nebraska Medical Center (Omaha, NE, USA). NALM6 cells were purchased from ATCC (Clone G5, CRL-3273). All cells were cultured in RPMI-1640 supplemented with 10% FBS and 1% penicillin/streptomycin at 37 °C in an incubator with a humidified atmosphere of 5% CO_2_ and 20% O_2_. All cell lines were authenticated by STR profiling and were determined to be free of mycoplasma contamination.

### Drugs and antibodies

The ITK inhibitor soquelitinib (CPI-818) were kindly provided by Corvus Pharmaceuticals Inc (CA). Brilliant Violet 510™ anti-human CD3 (#300448), PerCP/Cyanine5.5 anti-human CD4 (#300530), PerCP anti-human CD4 (#300528), APC/Cyanine7 anti-human CD8 (#344713), PE/Cyanine7 anti-human CD8 (#344750), FITC anti-human CD45RA (#304105), PE/Cyanine7 anti-human CD197 (CCR7) (#353225), BV421 anti-human CD25 (#356114), PE anti-human CD69 (#310906), PE anti-human CD244 (2B4) (#329507), FITC anti-human CD366 (TIM3) (#345022), FITC anti-human CD223 (LAG3) (#369308), PE anti-human TIGIT (#372704), APC anti-human CD279 (PD-1) (#379208), PE anti-human TNF-α(#502909), APC anti-human IFN-γ(#502512), PE anti-human CD107a (#328608), PE anti-human NFATc1 (#649605), PE anti-human BATF (#654803), PE anti-human IRF4 (#646403), FITC anti-human CD38 (#303504) and FITC anti-human CD39 (#328205) were purchased from Biolegend (San Diego, CA, USA). Rabbit Anti-Mouse FMC63 scFv Monoclonal Antibody (Alexa Fluor 647) from BioSwan Laboratories (#200102, Shanghai, China) was used to detect CAR-T cells. Phospho-Ser1248-PLCγ1 (#8713), PLCγ1 (#5690), TCF1 (#2203), NR4A (#3960), NFAT1 (#5861), Tox (#36778), BATF (#8638), TNF-a (#3707), LAG3 (#15372) and GAPDH (#5174) were provided by Cell Signaling Technology (Danvers, MA, USA) as a primary antibody for protein detection. Antibodies to IFN-γ were purchased from Proteintech Group (#15365-1-AP, Hubei, China). The secondary antibodies Anti-IgG (H + L chain) (Mouse, #330) pAb-HRP and Anti-IgG (H + L chain) (Rabbit, #458) pAb-HRP were purchased from Medical & Biological Laboratories Co., Ltd. (MBL, Tokyo, Japan).

### CAR-T cells preparation

Priming human CD3^+^ T cells were isolated from peripheral blood mononuclear cells (PBMCs) of healthy donors, and then activated by CD3/CD28 Dynabeads (T cells/beads = 1:1) (#11132D, Thermo Fisher, Waltham, MA, USA) in X-VIVO medium (# 04-418Q, Lonza, Basel, Switzerland) with 500 IU/mL human interleukin-2 (IL-2) (SL Pharm, Beijing, China). After 24 h, activated T cells were transduced with CD19 CAR encoding lentivirus and maintained at 1.5 × 10^6^ cells/mL in X-VIVO 15 media supplemented with 500 IU/mL hIL-2. Dynabeads were removed 3 days after transduction. During the cell culture, CAR-T cell proliferation and viability were tested every 2 days with automated cell counter (Countess 3 FL, Thermo Fisher, Waltham, MA, USA).

### Mouse Models

8-week-old female NCG mice from Beijing GemPharmatech were tail vein vaccination with mouse T cell lymphoma cell line NALM6-luc (5 × 10^6^ cells per mouse). A minimum of 6 animals per group was used, as this is the sample size allowing basic statistical tests (e.g., t-test, ANOVA) and consistent with guidelines for exploratory animal studies. Seven days after vaccination, CD19 CAR-T cells were reinfused into the tail vein, 1 × 10^6^ cells per mouse. Luciferase intensity in mice was measured on days 0, 3, 7, 10, and 14 after CAR-T reinfusion. Mice were assigned to groups using stratified balanced randomization based on their tumor burden on day 0, ensuring that each group had similar tumor burden and body weight. At the same time, the mortality of mice was recorded, and survival curves were drawn. Animals were excluded from the study if they presented with severe infection, suffered accidental death, or experienced body weight loss exceeding 20% of baseline. Investigators were blinded to group allocation during drug administration and subsequent outcome assessment, with complete blinding implemented. While mice were grouped via stratified balanced randomization (based on tumor burden and body weight on day 0 to ensure group comparability), the researchers who administered treatments (e.g., CAR-T cells) and assessed outcomes (e.g., tumor growth, survival, or functional readouts) remained unaware of the specific group assignments throughout these critical phases. This full blinding minimized potential observer bias in both intervention delivery and result evaluation. All animal experiments were performed according to the guidelines for the care and use of animals and were approved by the Peking University Cancer Hospital & Institute.

### Real-time PCR analysis

Total RNA was extracted using Trizol (Invitrogen, Waltham, MA, USA) according to the manufacturer’s instructions. On Applied Biosystems 7500-Fast devices, quantitative real-time PCR (qRT-PCR) was carried out using FastStart Universal Real-Time PCR Master Mix from TaKaRa. Huayu Gene created and synthesized the primers. The comparative Ct was adjusted to the GAPDH housekeeping gene, as shown below: ^Δ^Ct (sample) equals Ct (PD-1) minus Ct (GAPDH). Then, the relative expression folds compared to control were determined as follows: 2 - ^ΔΔ^Ct is 2^-(^Δ^Ct [sample] - ^Δ^Ct [control]).

### Flow cytometry analysis

CAR-T Cells were spun in polypropylene round-bottom tubes and resuspend in 100 µL PBS. Tubes were stained with antibodies, vortexed briefly and incubate cells at room temperature in the dark for 30 min. Antibodies were used at 5 µL/test unless otherwise indicated. Following antibody binding, tubes were washed twice with 1 mL PBS and spun down. Prior to flow cytometric analysis, cells were resuspended in 200 µL PBS and immediately analyzed on the Beckman Coulter CytoFLEX flow cytometer, and all flow cytometric analyses were performed using CytoFLEX built-in software and FlowJo (Becton Dickinson, Franklin Lakes, NJ Shiga, USA).

### Western blotting analysis

Sample preparation of whole cell lysates, SDS-PAGE, membrane transfer and blotting were performed according to standard protocols. Cells were lysed in cell lysis buffer (#INKLWB1000S, Invent, Beijing, China) supplemented with protease inhibitor cocktail tablets (#04693124001, Roche, Baden-Wuerttemberg, Germany) and phosphatase inhibitor cocktail tablets (#04906837001, Roche, Baden-Wuerttemberg, Germany). Protein concentration was determined using BCA Protein Assay Kit (#23227, Thermo Fisher, Waltham, MA, USA). 10–30 μg of protein was resolved by SDS-PAGE and transferred onto PVDF membranes. Samples were boiled in 95 °C for 10 min before loading. Then equivalent amounts of protein were separated by sodium dodecyl sulfate polyacrylamide gel electrophoresis (SDS-PAGE) on which separating gel contained 4–12% acrylamide (#NPO335BOX, Invitrogen, Carlsbad, CA, USA). Protein transferred to polyvinylidene fluoride (PVDF) membrane (#IPVH00010, Millipore Corporation, Billerica, MA, USA). Membranes were blocked and incubated overnight at 4 °C with primary antibodies, then incubate the secondary antibody for 1 h at room temperature. ECL select western blotting detection reagent (#WBKLS0500, Millipore Corporation, Billerica, MA, USA) was performed for detection using a chemiluminescence detection system (VILBER FUSION SL4, VILBER BIO IMAGING, Paris, France).

### Cytotoxicity assay

CAR-T cells treated with ITK inhibitor for 7 days were co-cultured with HBL-1 and DoHH2 cells for 5 h. The specific cytotoxicity of normal T cells was assessed using an LDH release assay using a CytoTox 96^®^ Non-Radioactive Cytotoxicity Assay (#G1780, Promega, Madison, WI, USA).

### ELISA

Use ELISA kit to detect TNF-α (#KIT10602, Sino Biological, Beijing, China) and IFN-γ (#1110002, Dakewe, Beijing, China) in the culture medium. Collect the culture medium after centrifugation and test according to the method specified in the instructions.

### Bulk RNA sequencing and data analyses

Total RNA extraction was performed for 1 × 10^6^ CD19 CAR-T cells derived from each experimental group using the FastPure cell/tissue total RNA isolation kit (CAT# RC112-01, Vazyme Biotech Co., Ltd., Beijing, China) according to the manufacturer’s instructions. Paired-end bulk RNA sequencing was performed to profile the transcriptomes. Raw sequencing reads were aligned to the GRCh38 primary reference genome using STAR (v2.7.10a), and transcript quantification was performed with featureCounts (v2.0.3) [40], using annotation from GENCODE v44. Raw expression values were normalized to Transcripts Per Kilobase Million (TPM) and visualized as heatmaps using the pheatmap package (v1.0.12) [41]. To assess sample reproducibility and variability, principal component analysis (PCA) was conducted to reduce transcriptomic dimensionality, and the first five principal components were used to perform UMAP visualization via the uwot package (v0.2.3). Differentially expressed genes (DEGs) were identified using the DESeq2 package (v1.46.0) with thresholds set at |log₂ fold change | > 1.2 and *P* < 0.05. Significant DEGs were visualized in volcano plots generated using ggplot2 [42]. KEGG pathway enrichment analysis of the DEGs was performed using the clusterProfiler package (v4.14.4), with upregulated and downregulated genes analyzed separately. Enriched pathways were ranked by p-value and presented as bubble plots. Gene Set Enrichment Analysis (GSEA) was conducted on TPM-normalized expression data using clusterProfiler (v4.14.4). The gene sets used included GSE9650_EXHAUSTED_VS_MEMO-RY_CD8_TCELL_UP, GSE44649_NAIVE_VS_ACTIV-ATED_CD8_TCELL_UP, PID_NFA-T_TFPATHWAY, and PID_TCR_CALCIUM_PATHWAY, obtained from the MSigDB database. GSEA was performed with 1,000 phenotype permutations to generate a null distribution of enrichment scores, from which normalized enrichment scores (NES) was calculated. Significantly enriched pathways were defined as those with |NES | > 1, *P* < 0.05, and *P*.adj < 0.25. In addition, for specific functional modules, Gene Set Variation Analysis (GSVA) (v2.0.5) was used to calculate enrichment scores across individual samples for downstream bioinformatic analyses.

### Statistical analysis

The sample size for each experimental group was set to a minimum of 3 (*n* ≥ 3) to ensure statistical validity for preliminary exploratory analyses (e.g., *t*-tests, ANOVA) and to detect meaningful trends in line with the study’s hypothesis. All statistical analyses were performed and visualized using GraphPad Prism software version 10.1.2 (GraphPad, San Diego, CA). Data distribution was assessed for normality prior to parametric testing. One-way ANOVA and Tukey’s test were performed to assess differences between groups treated with ITK inhibitor at various concentrations. Tumor growth data were analyzed with two-way ANOVA. Survival curves were analyzed by using a log-rank test. All statistical tests were two-sided. All values and error bars represent the mean ± SEM. In the figures, significance of findings was defined as follows: *P < 0.05; **P < 0.01; *** *P* < 0.001; ****P < 0.0001.

## Supplementary information

Supplementary Materials: S1-S3

Uncropped western blots

## Data Availability

The data for this study are available from the corresponding author upon reasonable request.
