# Supplementary material for: ITK-targeted immune remodeling enhanced the efficacy of anti-CD19 CAR-T cell therapy
Source: Cell Death Discov. 2026 Mar 6;12:131. doi: 10.1038/s41420-026-03004-2 (PMC13039719; doi:10.1038/s41420-026-03004-2)
Supplement: Supplementary file 1 — Supplementary Materials: S1-S3 [file 41420_2026_3004_MOESM1_ESM.docx]

Supplementary Materials for

**ITK-targeted immune remodeling enhanced the efficacy of anti-CD19 CAR-T cell therapy**

Zhenjun Li^1,#^, Liangcheng Lv^1,#^, Xiaoyu Yao^1,#^, Zhiwei Feng^1,#^, Yan Xie^2^, Kecheng Li^3^, Feifei Qi^4^, Mei Yang^1^, Jingwen Wang^1^, Tao Pan^1^, Xinghua Li^3^, Haiyan Chen^3^, Jing Wang^4^, Yanping Ding^4^, Jun Zhu^2^, Yuqin Song^2^, Xiaomin Wang^1,*^, Ning Ding^1,*^.

1 Key laboratory of Carcinogenesis and Translational Research (Ministry of Education), Laboratory of Lymphoma Translational Research, Peking University Cancer Hospital & Institute. No. 52 Fucheng Rd, Haidian District, Beijing, 100142, China

2 Key laboratory of Carcinogenesis and Translational Research (Ministry of Education), Department of Lymphoma, Peking University Cancer Hospital & Institute. No. 52 Fucheng Rd, Haidian District, Beijing, 100142, China

3 Angel Pharmaceuticals Co., Ltd. Jiaxing, Zhejiang Province, China

4 Beijing Imunopharm Technology Co. Ltd. Beijing, China

These authors contributed equally: Zhenjun Li, Liangcheng Lv, Xiaoyu Yao, Zhiwei Feng

*Correspondence: wangxiaomin@bjmu.edu.cn; ningding@bjmu.edu.cn

**This file includes:**

Supplementary Figures: S1-S3


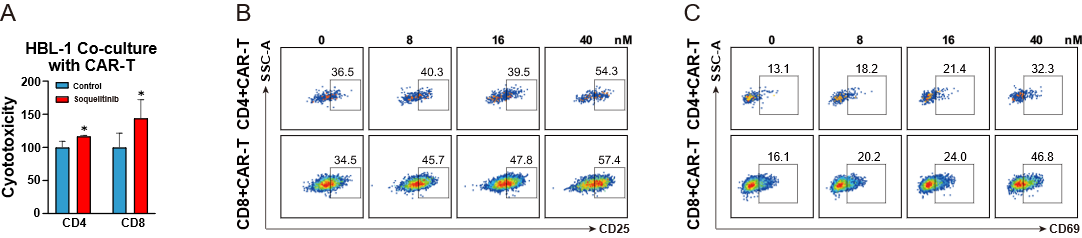


**Supplementary Fig. 1 Soquelitinib enhances the activation and cytotoxic activity of CD19 CAR-T cells.**

(A) CAR-T cells were treated with soquelitinib or vehicle control, after which CD4+ and CD8+ subsets were isolated using fluorescence-activated cell sorting (FACS). The CD4+ and CD8+ subsets were then individually assessed for their cytotoxic activity against HBL-1 target cells. Coculture duration: 6h; E:T ratio=4:1; n=3. (B) Representative flow cytometry contour plots of CD25+ cells in CD4⁺ (upper) and CD8⁺ (lower) CAR-T subpopulations. (C) Representative flow cytometry contour plots of CD69+ cells in CD4⁺ (upper) and CD8⁺ (lower) CAR-T subpopulations.


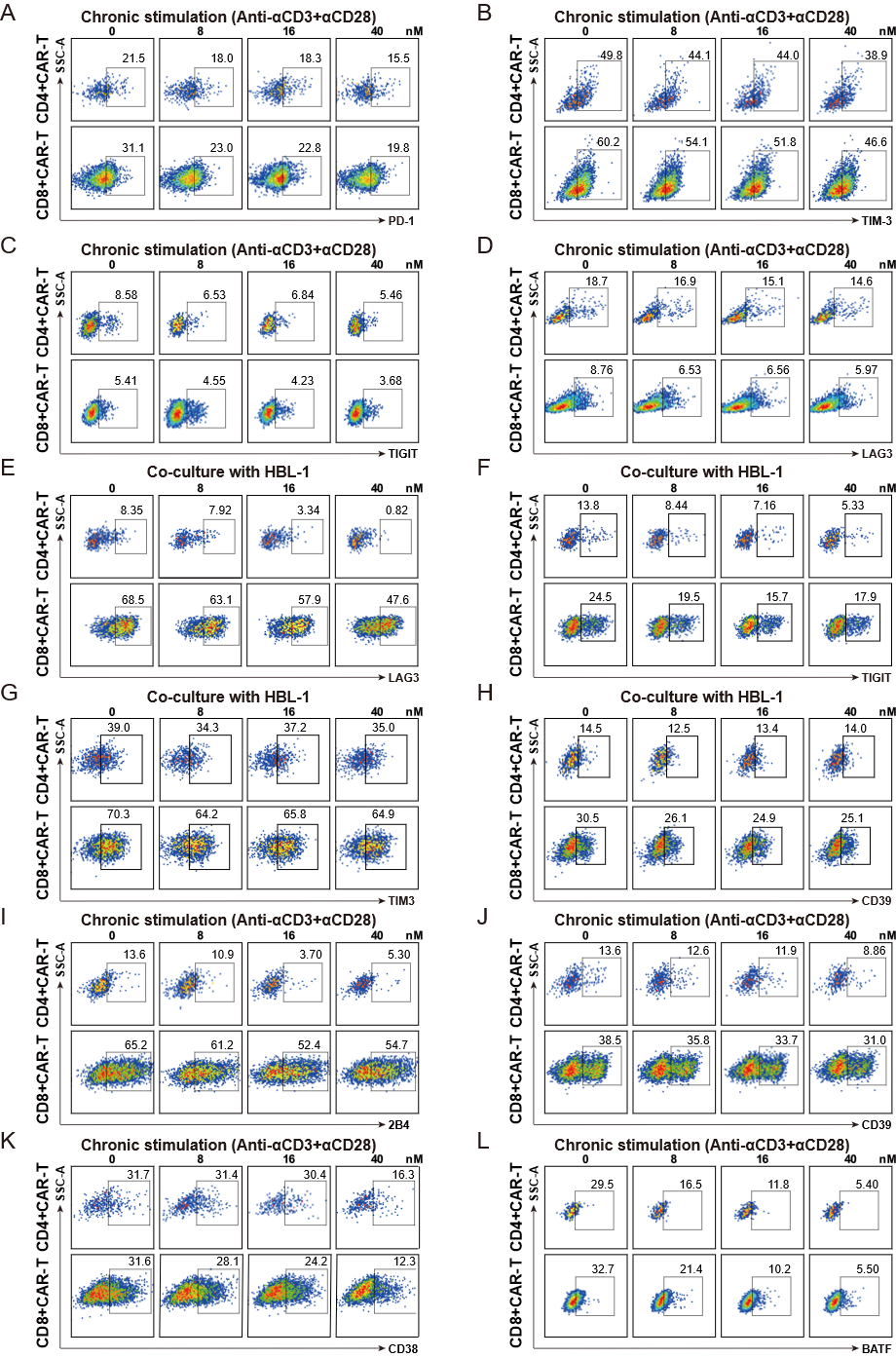


**Supplementary Fig. 2 Soquelitinib effectively reduces the exhaustion of CD19 CAR-T cells in**

(A-D) Representative flow cytometry plots of PD-1+, TIM-3+, TIGIT+, and LAG-3+ cells within CD4+ and CD8+ CD19 CAR-T cell populations. (E-H) Representative flow cytometry plots of LAG3+, TIGIT+, TIM3+, and CD39+ cells within CD4+ and CD8+ CD19 CAR-T cell populations. (I-K) Representative flow cytometry plots of 2B4+, CD39+ and CD38+ cells within CD4+ and CD8+ CD19 CAR-T cell populations. (L) Representative flow cytometry plots of BATF+ cells within CD4+ and CD8+ CD19 CAR-T cell populations.


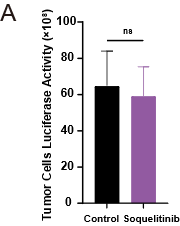


**Supplementary Fig. 3 Soquelitinib does not exhibit direct antitumor activity.**

(A) A statistical analysis of tumor bioluminescence signals was performed, comparing mice treated with Soquelitinib alone and those receiving vehicle control only. Tumor burden quantification: Bioluminescent flux (scale: 10⁸ photons/sec/cm²/sr) measured in regions of interest (ROI) following IVIS imaging at indicated timepoints; n=6.
