# Supplementary figures and images for: ITK-targeted immune remodeling enhanced the efficacy of anti-CD19 CAR-T cell therapy
Source: Cell Death Discov. 2026 Mar 6;12:131. doi: 10.1038/s41420-026-03004-2 (PMC13039719; doi:10.1038/s41420-026-03004-2)

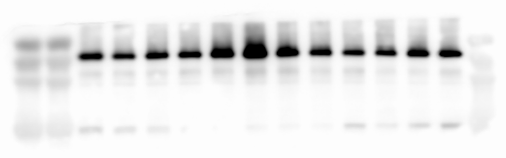
**
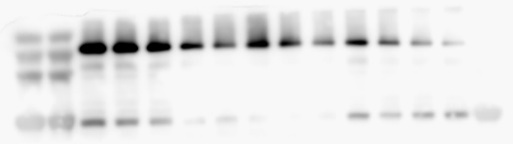
Figure 4M**

PLCγ-1

p-PLCγ-1


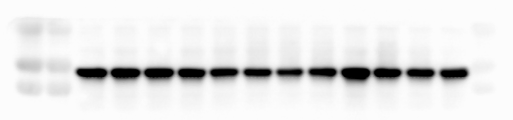


GAPDH

**Figure 4N**













GAPDH

TOX

NFAT1

NR4A

TCF1





BATF

**Figure 4O**

**

**





TNF-α

IFN-γ


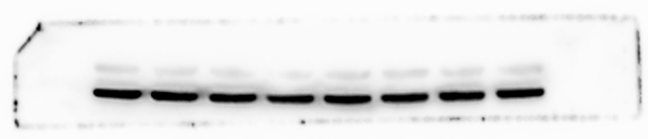




GAPDH

LAG3

Supplement: Supplementary file 2 — Uncropped western blots [file 41420_2026_3004_MOESM2_ESM.docx]
